# Healthier SG: for a healthier Singapore and beyond

**DOI:** 10.1016/j.lanwpc.2023.100893

**Published:** 2023-08-31

**Authors:** The Lancet Regional Health – Western Pacific

On July 5, 2023, the Singapore government officially rolled out the Healthier SG, a health-care reform plan that shifts the country's health-care delivery strategy from curative care to preventive care. According to the white paper on Healthier SG released on September 21, 2022, residents are encouraged to enrol with a family doctor who will work with community partners, regional health managers, and other sectors to provide a life-course and whole-person health-care service. The programme will start to enrol people aged 60 years and older and will expand to those aged 40–59 years later.

Singapore is a city-state with 5.64 million people of Chinese, Eurasian, Indian, and Malay backgrounds. It is among the countries with the highest health-adjusted life expectancy at birth. Despite declines due to mortality in the COVID-19 pandemic, the life expectancy at birth is stable at 80.7 years for men and 85.2 years women in 2022. These health achievements are mainly attributed to the high-quality health-care system that is accessible to all citizens in the country. Although the health-care system is efficient at delivering curative care, there is a large unmet need for primary care service. For instance, clinics run by private general practitioners (GPs) provide around 80% of primary care service, but many of them do not have the resources to manage complex medical needs, while government funded polyclinics could provide those medical services but are often too crowded. The rapidly ageing population and high burden of non-communicable diseases are driving escalating demands for medical services and increasing pressures on the health-care system, with rising needs for public hospital beds and long-term care services, resulting in a higher health spending. As such, Healthier SG represents an evolution of the health-care system from the current curative care system to integrated, longitudinal, and community-based care.

The strength of Healthier SG reform is to value the crucial role of GPs in delivering life-course and whole-person preventive care to the public. As the reform substantially expands the scope of GPs’ duties, Chuan De Foo and colleagues suggested the government should strengthen primary care and engage family doctors with administrative support, skills training, and digital solutions, along with fostering trust among different sectors. The government also needs to find approaches to expand the numbers of health-care workers and raise the profile of family medicine through education reform. While the public was involved in the policy making process, public engagement should be prioritised in the implementation stage of Healthier SG by means of patient advisory councils and quality improvement teams to optimise uptake and effective implementation at the local level.

Another welcomed aspect of Healthier SG is that it addresses the social determinants of health. The Singapore health-care system emphasises personal responsibility for health, but this should not overshadow structural issues. The Singapore Minister of Health, Ong Ye Kung, addressed in a meeting in 2022 that population ageing and social inequality are the two big social challenges in the country. Social inequality might lead to health inequality as people with lower socioeconomic status usually have worse health outcomes. In the white paper on Healthier SG, social prescribing was recommended to link individuals to the community and support positive lifestyle and behaviour changes to address social determinants of health. Although highlighted at the policy level, local research on social prescribing and its impact on health and wellbeing are limited. Kheng Hock Lee and colleagues have led a social prescribing programme for older adults in SingHealth Community Hospitals since 2019. They summarise their lessons learnt from the intervention design to the implementation phase, which provides valuable information and guidance on incorporating social prescribing as part of Healthier SG. In time, similar to the research agenda developed by Sahil Sandhu and colleagues for social prescribing in the UK, Singapore will also benefit from having guidelines on how to assess patients' social needs, designing frameworks to evaluate the effectiveness of interventions, and understanding internal and external factors affecting the implementation and sustainability of interventions. These approaches will eventually provide solid evidence for policy making for the country.

In a letter published in *The Lancet*, Tikki Pang said "Singapore has much to offer in terms of knowledge and innovations". Singapore ranked tenth in the 2022 World Index of Healthcare Innovation; it was praised for its robust strategic response towards the COVID-19 pandemic and showed a good model to mitigate the spread of the virus for the region and globally. Despite still being in its early stages, we hope Healthier SG will be another good example that could benefit our region and countries facing population ageing and rising burdens of non-communicable diseases.

